# Challenging Extraction and Replacement of an Eight-year-old Nanostim Leadless Pacemaker and AVEIR Implant

**DOI:** 10.19102/icrm.2024.15126

**Published:** 2024-12-15

**Authors:** Evan Czulada, Rajiv Kabadi, Binaya Basyal, Cyrus Hadadi, Athanasios Thomaides

**Affiliations:** 1 Georgetown University School of Medicine, Washington, DC, USA; 2 Department of Electrophysiology, MedStar Health: Heart and Vascular Institute at MedStar Washington Hospital Center, Washington, DC, USA

**Keywords:** Cardiac implantable electronic device management, high-grade AV block, lead extraction, leadless pacemaker, permanent atrial fibrillation

## Abstract

Leadless pacemakers (LPs) are emerging options for bradyarrhythmias. However, extraction can be risky if the device is in an unfavorable position. We present a challenging case of a Nanostim LP (NLP) (Abbott Medical Inc., Abbott Park, IL, USA) placed 8 years prior to removal and subsequent replacement with an AVEIR LP (ALP) (Abbott). A 72-year-old man received an NLP in 2015 for persistent atrial fibrillation with bradycardia. The NLP could not be interrogated in our office. An external event monitor demonstrated persistent atrial fibrillation with bradycardia and pauses. A premature battery depletion of the NLP was suspected. An ALP was chosen for replacement. On a computed tomography scan of the chest, the NLP was seen in the mid-free wall of the right ventricle, and its proximal portion abutted the tricuspid annulus. The AVEIR retrieval catheter (ARC) was used for retrieval. Multiple attempts were made to snare the device, yet it proved difficult due to poor placement and button tissue formation. The snare became damaged, and a second ARC was needed. On the successful attempt, the NLP was snared proximally and permitted docking. We advanced the protective sleeve over the NLP body, but resistance was observed due to tissue growth. Counterclockwise torsion was applied, and the device disconnected. Once the NLP was in linear orientation, the protective sleeve was eventually positioned over its body, allowing removal. The ALP was then installed without difficulty or structural complications. This report shows the importance of proper LP placement in the right ventricular septal wall. LP removal can be performed safely, yet complications can arise based on the age and location of the LP. The ARC can be successfully used to extract non-AVEIR LPs with evidence of docking button tissue growth. Similar interventions should exercise caution when attempting extraction and subsequent implantation.

## Case presentation

A 72-year-old man was diagnosed with atrial fibrillation and symptomatic bradycardia and received a Nanostim leadless pacemaker (NLP) (Abbott Medical Inc., Abbott Park, IL, USA) in 2015. Based on a previous electrocardiogram, this leadless pacemaker (LP) was suspected to have been set to VVI 60 bpm; yet, upon follow-up, it was unable to be interrogated. An external event monitor was placed, which demonstrated an average heart rate of 45 bpm with long pauses and intermittent pacing at 30 bpm. Accordingly, the patient was determined to be ideally suited for an AVEIR LP (ALP) (Abbott) based on the need for ventricular pacing due to bradycardia with long-standing permanent atrial fibrillation. We discussed standard transvenous VVI pacemaker versus removal and replacement of the NLP with an ALP. We explained that both NLP and ALP were specifically designed for the latter. The benefits and risks of the procedure versus device abandonment were discussed with the patient, who elected to undergo removal and replacement.

**Figure 1:**
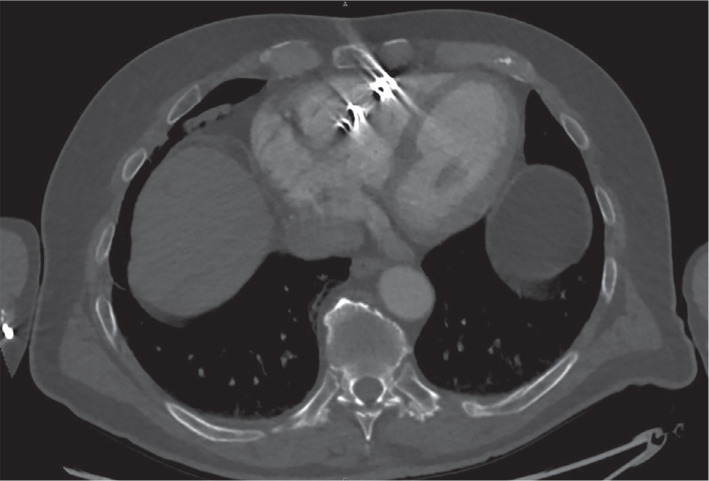
Visualization of the Nanostim leadless pacemaker on a computed tomography scan of the chest. The patient’s preoperative chest computed tomography scan showed that the previously implanted Nanostim leadless pacemaker was positioned in the lateral free wall of the right ventricle, adjacent to the tricuspid annulus.

**Figure 2:**
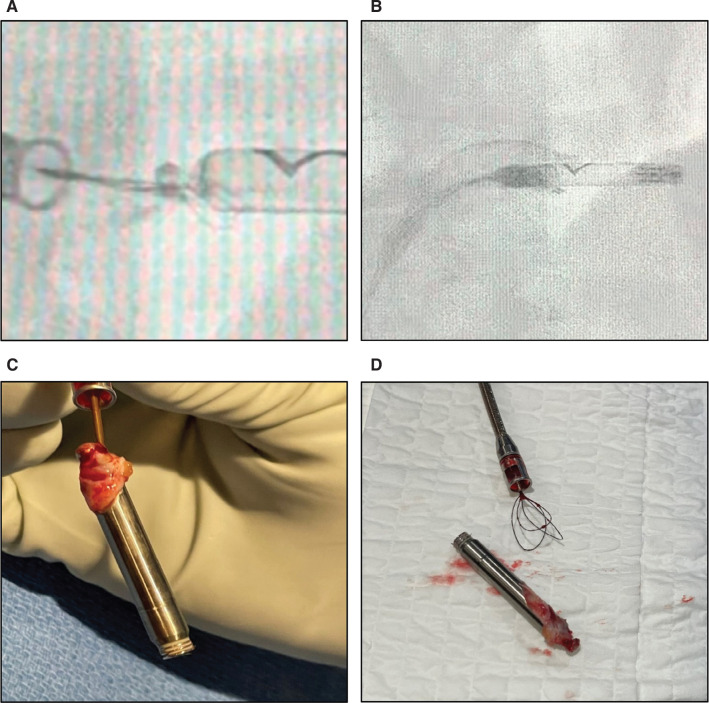
Evidence of the Nanostim leadless pacemaker’s challenging removal. **A:** A zoomed-in view of the remaining snare loop around the device following unsuccessful snaring. **B:** The resultant protective sleeve ballooning upon retrieval with the AVEIR retrieval catheter. **C:** Extracted pacemaker demonstrating extensive adhesions and taut fibrous tissue on its proximal end due to poor placement in the right ventricular free wall. **D:** Extracted device with unfurled fibrosis next to the used snare of the second AVEIR retrieval catheter.

A preoperative computed tomography scan of the patient’s chest demonstrated the NLP in the mid-right ventricular (RV) free wall (RVFW; **Figure 1**) with its proximal end abutting the tricuspid annulus (TA). Intraoperative imaging under fluoroscopy confirmed the NLP’s lateral placement on the RVFW and TA. Femoral venous access was granted using a 27-French (Fr) sheath, which was advanced into the right atrium. Retrieval was accomplished using an AVEIR retrieval catheter (ARC). A right ventriculogram was performed with contrast injected via a 5-Fr vein selector (Merit Medical, South Jordan, UT, USA). Right (20°) and left (30°) anterior oblique projections were chosen for extraction. Multiple attempts were made to snare the device, yet snaring proved difficult due to poor placement in the RVFW and TA proximity. We snared the Nanostim button at least five times, but the device kept slipping off every time we attempted to dock due to tissue formation over the button **(Figure 2A)**. The snare subsequently became worn and damaged, necessitating a second ARC. On the final attempt, the NLP was snared closer to the proximal tip. This provided more stability during alignment and allowed docking. We attempted to advance the protective sleeve over the Nanostim body as recommended. However, we observed significant resistance due to tissue growth on the proximal LP and decided to halt any further progress **(Figure 2B)**. Counterclockwise torsion was applied via the ARC, and the device disconnected from the myocardium. The system was withdrawn to the right atrium. Once the LP was in linear orientation with the ARC, we positioned the protective sleeve over the entire LP body and removed it. The LP inspection was notable for extensive fibrotic tissue overlaying the button and proximal device that likely contributed to its difficult removal **(Figures 2C and 2D)**. The distal end and helix-fixation apparatus remained free of tissue.

Using the same 27-Fr sheath, an ALP was advanced and deployed on the RV septum under fluoroscopy. Gentle manual tension on the tether demonstrated adequate myocardial contact. The new device sensing was 4.5 mV, the impedance was 540 Ω, and the pacing threshold was 1.25 V at 0.4 ms, with the device programmed to VVI 50 bpm. After testing the ALP, the tether was removed, and the device was deployed. A transesophageal echocardiogram confirmed no evidence of tricuspid valve injury or tricuspid regurgitation, and the catheter and the sheath were removed. After the procedure, the patient returned to the recovery area in a stable condition and was symptom-free at his follow-up appointment.

The patient provided informed consent for the publication of this case report.

## Discussion

Due to the many established complications from conventional LP systems,^1^ LPs are emerging as a preferred alternative for the treatment of bradyarrhythmia. Despite their promise, LPs present their own challenges, and one of the most documented examples was the premature battery failure and subsequent recall of the NLP.^2^ Experiences with NLP extraction have been mostly successful, and the only known issues have been related to arteriovenous fistula or pseudoaneurysm formation and docking button detachment.^2–6^ Here, we present a unique, challenging case of an NLP placed 8 years prior to removal and subsequent replacement with an ALP, the first of its kind in the United States.

The oldest known NLP extraction case (9 years) was recently reported from the Czech Republic.^7^ Despite the device’s great age, Neužil et al. (2023) experienced little difficulty in its retrieval, citing only 2 min from the catheter entrance into the RV to device removal, likely due to the NLP’s proper placement in the RV mid-septum. Indeed, the risk of these procedures is magnified when the device is placed in a suboptimal position, such as the RVFW. RVFW deployment of NLPs increases extraction difficulty and has been implicated in some cases of tricuspid regurgitation during and after removal.^2,8,9^ While many operators opt to abandon these difficult-to-reach devices, doing so may increase long-term infection risk. Furthermore, leaving behind superfluous hardware while adding new LPs with currently unknown battery life can lead to multiple device implantations and potentially disastrous consequences over time.

While the NLP’s helix-fixation technology was originally designed to expedite acute and chronic removal, its extraction can evidentially become complicated. Specifically, this case adds evidence to the observed obstacles associated with pacemaker extraction near the TA in the RVFW compared to the recommended RV septum placement.^8^ NLP extraction is achieved by snaring the docking button found on the device’s proximal end, yet accessing this mechanism becomes ambitious when it is blocked by the TA or becomes overgrown with fibrotic tissue, which often necessitates device abandonment.^9^ In our case, multiple consecutive attempts were necessary to ensnare and dock the LP because of fibrosis overlaying the button and proximal LP as well as the proximity of the LP tail to the TA. Even still, the proximal part of the NLP was moving freely and was not tethered to the RV or TA, as evidenced by our ability to pass the snare around it.

Moreover, we extracted the older NLP using the ARC, which had been initially designed for the ALP. Procedural success may have been predicated on using the ARC to twist the tissue on itself, and the new tissue tautness could have freed up space for the snare on the docking button. This is shown in the difference between the hold **(Figure 2C)** and subsequent release **(Figure 2D)** of the native RV or TA remnants on the NLP. Another challenge during this extraction was our inability to advance the protective sleeve over the full LP body **(Figure 2B)**. The manufacturer recommends this step to confirm that no tissue is trapped between the device and the docking cap while simultaneously covering the fixation helix after it is unscrewed. If protective sleeve billowing is seen, the manufacturer recommends undocking the device, redocking it, and then readvancing the protective sleeve. Clearly, with this device, we were unable to advance the protective sleeve due to the presence of significant tissue growth. There is no known way to remove this tissue endovascularly to extract the LP more efficiently.

While our patient developed no significant changes in cardiovascular function during or following removal, tissue overgrowth was grossly visualized on the device’s proximal end **(Figures 2C and 2D)**. The lack of tissue on the device’s distal, helix-fixating end suggests that this material was the result of fibrosis rather than myocardium. Given that the proximal end of the NLP had made contact with the basal RV or TA, endothelium and subsequently thick fibrotic tissue formed on the NLP over its 8-year implantation history. This extraction difficulty was not replicated with implantation of the ALP, thus implying an increased risk upon removal—rather than replacement—of older, positionally complex devices due to intact myocardium underlying the properly placed ALP in the RV septum.

## Conclusions

Combined with previous evidence, this case report of an 8-year-old LP extraction and exchange highlights the importance of proper LP placement in the RV septal wall, especially as more of the aging NLPs fail due to battery depletion. Reports of the removal and replacement of these devices provide documented evidence of safety, yet complications can arise based on the age and location of the LP. For LPs implanted in the RVFW, the ARC can be successfully used by leveraging the extraction catheter angle, which is increased when its posterior end points inferiorly. The ARC can also tighten any tissue covering the docking button, thus creating space for the snare and permitting subsequent extraction. Future interventions in patients with similar devices should exercise caution when attempting extraction and subsequent implantation of newer models.
